# Dopamine D1 receptor activation regulates the expression of the estrogen synthesis gene aromatase B in radial glial cells

**DOI:** 10.3389/fnins.2015.00310

**Published:** 2015-09-02

**Authors:** Lei Xing, Heather McDonald, Dillon F. Da Fonte, Juan M. Gutierrez-Villagomez, Vance L. Trudeau

**Affiliations:** Department of Biology, Centre for Advanced Research in Environmental Genomics, University of OttawaOttawa, ON, Canada

**Keywords:** radial glial cell, dopamine, aromatase, neurogenesis

## Abstract

Radial glial cells (RGCs) are abundant stem-like non-neuronal progenitors that are important for adult neurogenesis and brain repair, yet little is known about their regulation by neurotransmitters. Here we provide evidence for neuronal-glial interactions via a novel role for dopamine to stimulate RGC function. Goldfish were chosen as the model organism due to the abundance of RGCs and regenerative abilities of the adult central nervous system. A close anatomical relationship was observed between tyrosine hydroxylase-positive catecholaminergic cell bodies and axons and dopamine-D1 receptor expressing RGCs along the ventricular surface of telencephalon, a site of active neurogenesis. A primary cell culture model was established and immunofluorescence analysis indicates that *in vitro* RGCs from female goldfish retain their major characteristics *in vivo*, including expression of glial fibrillary acidic protein and brain lipid binding protein. The estrogen synthesis enzyme aromatase B is exclusively found in RGCs, but this is lost as cells differentiate to neurons and other glial types in adult teleost brain. Pharmacological experiments using the cultured RGCs established that specific activation of dopamine D1 receptors up-regulates aromatase B mRNA through a cyclic adenosine monophosphate-dependent molecular mechanism. These data indicate that dopamine enhances the steroidogenic function of this neuronal progenitor cell.

## Introduction

The radial glial cell (RGC) is a cell type in the central nervous system (CNS) of all vertebrate species. As the key organizer and contributor of both nervous system development and adult neurogenesis, RGCs function as scaffolding to support neuron migration (Schmechel and Rakic, 1979; Bentivoglio and Mazzarello, 1999), and as stem cells that give rise to both neurons and other glial cells (Zupanc and Clint, 2003; Zupanc et al., 2012). Despite these essential functions, RGCs are transient in mammals as the majority differentiate into other cell types during the developmental process (Malatesta et al., 2000; Rakic, 2003). In comparison, RGCs are highly abundant and persistent throughout the entire lifespan of fish, explaining the high regenerative capacity of the teleost CNS. To maintain normal brain function, regulation and termination of the regenerative process is critical. Therefore, RGCs must be under tight control by specific regulators to prevent the overproduction of newborn cells.

Studies in mammalian model systems indicate that neurotransmitters such as GABA and dopamine (DA) influence neurogenesis by modulating stem and progenitor cell functions (Höglinger et al., 2004; Kippin et al., 2005; Ge et al., 2007). Our earlier work was suggestive of DA-glial interaction since we observed rapid *in vivo* effects of DA receptor selective pharmacological agents on glial fibrillary acidic protein (GFAP) expression in goldfish hypothalamus (Popesku et al., 2010). In zebrafish, a close anatomical relationship exits between RGCs and serotonin neurons in the hypothalamic paraventricular organ (Pérez et al., 2013); serotonin being another potential neurotransmitter that may regulate this critical cell type, although specific receptors or functions for serotonin or any other neurotransmitter in RGCs have not yet been identified.

Here we take advantage of the high abundance of RGCs in goldfish, and have investigated the anatomical relationship between DA neurons and RGCs and developed an *in vitro* culture system to specifically investigate DA signaling pathways. The teleost RGC also represents the only native cell model to date that allows the distinction between glial and non-glial estrogen because it is the unique cell type in the adult teleost brain that produces the estrogen synthesis enzyme aromatase B (*cyp19a1b*) in normal brain or after damage (Forlano et al., 2001; Diotel et al., 2010a). Aromatase B *(cyp19a1b)* expression is lost as RGCs differentiate into neurons, therefore the overexpression of aromatase B is a key signal for maintaining the progenitor characteristics of this cell type (Diotel et al., 2010a; Xing et al., 2014; Coumailleau et al., 2015). Aromatase B is the specific marker and functional target herein investigated.

## Materials and methods

### Experimental animals

All procedures used were approved by the University of Ottawa Protocol Review Committee and followed standard Canadian Council on Animal Care guidelines on the use of animals in research. Common adult female goldfish *(Carassius auratus)* were maintained at 18°C under a natural simulated photoperiod and fed standard goldfish flakes. Sexually mature female goldfish (18–35 g) were anesthetized using 3-aminobenzoic acid ethylester (MS222) for all handling and dissection procedures.

### Cell culture

Here we focus on females to follow previous large-scale transcriptomic and proteomic analyses of the female goldfish brain (Zhang et al., 2009; Popesku et al., 2010). The hypothalamus and telencephalon were dissected from female goldfish and minced into small pieces. Radial glial cells were dissociated with trypsin (0.25%, Gibco) and cultured in Leibovitz's L-15 medium (Gibco) with 15% Fetal Bovine Serum (FBS, Gibco) and antibiotic-antimycotic solution. Cell culture medium was changed once a week after initial isolation. Radial glial cells were subcultured by trypsinization (0.125%) for 3 passages and then used for experiments.

### Immunohistochemistry

After overnight fixation, female goldfish brains were washed and embedded in Shandon Cryomatrix (Thermo Scientific), snap frozen in liquid nitrogen, and kept at −80°C. Frozen blocks were sectioned and blocked in blocking buffer (0.3% Triton PBS containing 1% bovine serum albumin (BSA) for 45 min at RT. Brain section and fixed cell slides were covered with the anti-GFAP (mouse, 1:800, Millipore, MAB360) (Forlano et al., 2001), anti-aromatase B (rabbit, 1:800, from the lab of O. Kah (Menuet et al., 2003; Pellegrini et al., 2007), anti-D1R antibody (rabbit, 1:500, Acris, AP09962PU-N), or anti-TH antibody (rabbit, 1:500, Abcam, AB152) (Yamamoto et al., 2011) then incubated with donkey anti-rabbit Alexa fluor 488 (1:500, Molecular Probes) and goat anti-mouse Alexa fluor 596 (1:500, Molecular Probes) for 1 h at RT. Slides were washed and mounted with the antifading medium Vectashield with 4,6-diamino-2-phenylindole (DAPI). Images were taken by Nikon A1RsiMP confocal microscope with Nikon's Imaging Software NIS-Elements. Neuroanatomical nomenclature follows Peter and Gill (Bannerman et al., 2006).

### *In situ* hybridization

Antisense RNA probes were synthesized *in vitro* using goldfish aromatase B cDNA fragments (1.6 kb). *In situ* hybridization on cultures was performed as previously described with minor modifications (Smith et al., 2007). Briefly, cells were fixed and incubated with probe mix in an oven at 70°C overnight, and blocked in 10% calf serum for 1 h at RT. Anti-DIG AP Fab fragment antibody (Roche) was added and incubated overnight at 4°C, then incubated with NTMT containing BCIP and NBT overnight at RT.

### RNA extraction, cDNA synthesis and quantitative real-time RT-PCR

Cells were exposed to selective D1 agonist (SKF 38393, Tocris) and selective D2 agonist (Quinpirole, Tocris) for 24 h at various concentrations to study the dose-dependent effects on *cyp19a1b* mRNA. To investigate DA receptor activation, cAMP and PKA involvement in *cyp19a1b* mRNA regulation by SKF 38393, cells were pre-exposed to 5 μM Flupentixol (Tocris), 100 μM SKF 83566 (Tocris), 100 μM DOM (Sigma), or 10 μM H89 (Tocris) for 1 h, then exposed to 10 μM SKF 38393 and 8-Br-cAMP (Tocris) for 24 h. The isolation of total RNA was performed by using RNeasy Micro kit (Qiagen). Total cDNA was prepared using Maxima cDNA synthesis kit (Thermo Scientific) and used as the template for the real-time RT-PCR assays. Primers used in the present study were designed using Primer3 (http://primer3.sourceforge.net/) and synthesized by Invitrogen (cyp19a1bFw, TGCTGACATAAGGGCAATGA; cyp19a1bRv, GGAAGTAAAATGGGT TGTGGA; 18sFw, AAACGGCTACCACATCCAAG; 18sRv, CACCAGATTTGCCCT CCA). The Maxima SYBR green qPCR Master Mix (Thermo Scientific) and CFX96 Real-Time PCR Detection System (Bio-Rad) were used to amplify and detect the transcripts of interest. Data were analyzed using the Bio-Rad software package. The relative standard curve method was used to calculate relative mRNA abundance between samples based on the Cq values, which were then normalized by using NORMA-GENE algorithm (Heckmann et al., 2011). Normalized data were used to calculate fold-change against the average value of control and then presented as means + SEM of gene expression from 4 biological replicates (*n* = 4) (assayed in duplicate) for each group.

### Measurement of cAMP production

To examine the induction of cAMP production by DA compounds in RGC culture, RGCs were seeded in 25 cm^2^ flasks and pre-incubated with IBMX (0.1 mM, Sigma) for 15 min, then treated with SKF 38393 (10 μM) for 30 min. Cell culture medium and cell extract were quantified with a cyclic AMP select EIA Kit (Cayman, 501040) according to the manufacturer's instructions.

### Western blot

Cells were exposed to 10 μM SKF 38393 for 24 h to study phosphorylated cAMP binding protein (p-CREB) stimulation. Total protein was denatured and separated by electrophoresis on a 10% SDS–polyacrylamide gel and transferred to a PVDF membrane as described previously (Zhao et al., 2006). Membranes were incubated separately with primary anti-actin (Cedarlane, CLT9001) and anti-Phospho-CREB (Ser133, Cell Signaling Technology, 9198S) antibodies overnight at 4°C and then incubated separately with goat anti-mouse IgG-HRP (Santa Cruz, sc-2005), and donkey anti-rabbit IgG antibody (GE Health Care, NA934VS). Blots were visualized using the Amersham ECL Prime Western Blotting Detection System (GE Health Care, RPN2232). Images were obtained using a Fusion FX5 and analyzed with Image J.

### Statistics

Data are presented as mean + SEM. Comparison of more than two groups was performed using One-Way analysis of variance (ANOVA) or Two-Way ANOVA followed by Tukey's *post-hoc* test or Dunnett's *post-hoc* test if the data did not pass homogeneity test in IBM SPSS Statistics Version 22. Comparison of two groups was performed using Student's *t*-test in IBM SPSS Statistics Version 22.

## Results

### There is a close anatomical relationship between catecholaminergic neurons and aromatase B-positive RGCs

**Figure 1:**
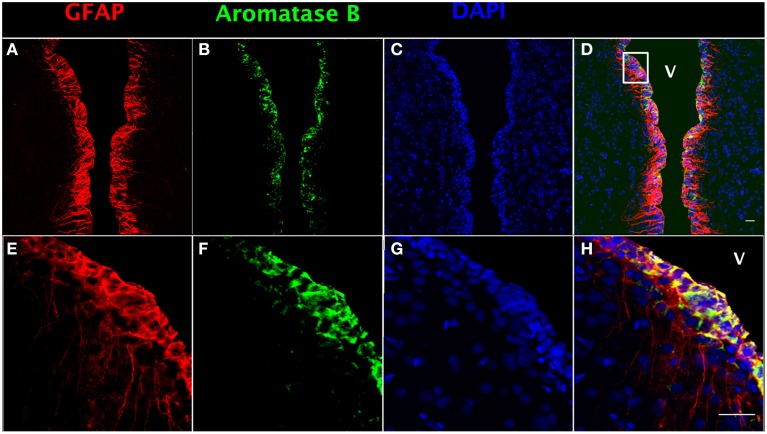
**Double fluorescence detection of GFAP (red; A,E) and aromatase B (green; B,F) along the ventricular surface in female goldfish telencephalon**. The confocal images show expression of both aromatase B and GFAP along the ventricular surface (V; ventricle). The merged images show colocalization of aromatase B and GFAP in RGCs **(D,H)**. The nuclear stain DAPI (blue) is also shown **(C,G)**. Scale bar = 20 μm.

In order to localize aromatase B protein in goldfish telencephalon, double immunofluorescence detection was performed. GFAP was used as a RGC cell marker (Zupanc and Sîrbulescu, 2011) to identify RGCs in female goldfish brain (Figures 1A,E), combined with the aromatase B antibody (Figures 1B,F) and DAPI (Figures 1C,G). The yellow color in double fluorescence detection showed that aromatase B was strongly expressed by the majority of GFAP-positive RGCs along the ventricular surface (Figures 1D,H).

**Figure 2:**
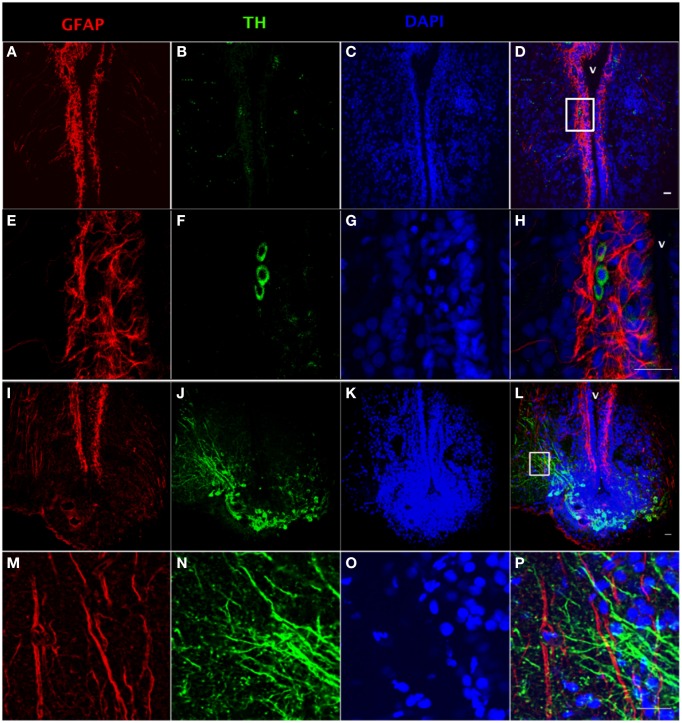
**Double fluorescence detection of GFAP (red) and TH (green) in ventral telencephalic area of the female goldfish brain**. The confocal images show RGC cell bodies marked by GFAP located along the ventricular surface **(A,E)**. Some catecholaminergic neuron cell bodies marked by TH are located along the ventricular surface **(B,F)**. Magnification of the boxed area in **(D)** shows that there are very close anatomical relationships between RGC fibers and catecholaminergic cell bodies **(H)**. The confocal image shows RGC cell bodies marked by GFAP located along the ventricular surface with fibers extending from ventricular surface to the area ventralis telencephali pars ventralis **(I,M)**. Catecholaminergic neuron cell bodies marked by TH are located at preoptic periventricular nucleus (NPP) with fibers extending to the area ventralis telencephali pars ventralis **(J,N)**. Magnification of the boxed area in **(L)** shows that there are very close anatomical relationships between catecholaminergic neuronal processes and RGC fibers (V; ventricle, **H,P**). The nuclear stain DAPI (blue) is also shown **(C,G,K,O)**. Scale bar = 20 μm.

Using tyrosine hydroxylase (TH) and GFAP double fluorescence detection, we show that there is a close anatomical relationship between catecholaminergic neuronal cell bodies and RGC fibers (Figures 2C,D,G,H) and between catecholaminergic neuronal processes and RGC fibers (Figures 2K,L,O,P). Intensive TH-positive cell bodies were observed along the ventricular surface (Figures 2B,F) and in the preoptic periventricular nucleus with fibers extending to the area ventralis telencephali pars ventralis (Vv) (Figures 2J,N). GFAP-immunoreactive RGCs were observed along the ventricular surface with fibers extending to the area ventralis telencephali pars ventralis in telencephalon (Figures 2A,E,I,M).

### Expression of dopamine D1 receptor in RGCs

**Figure 3:**
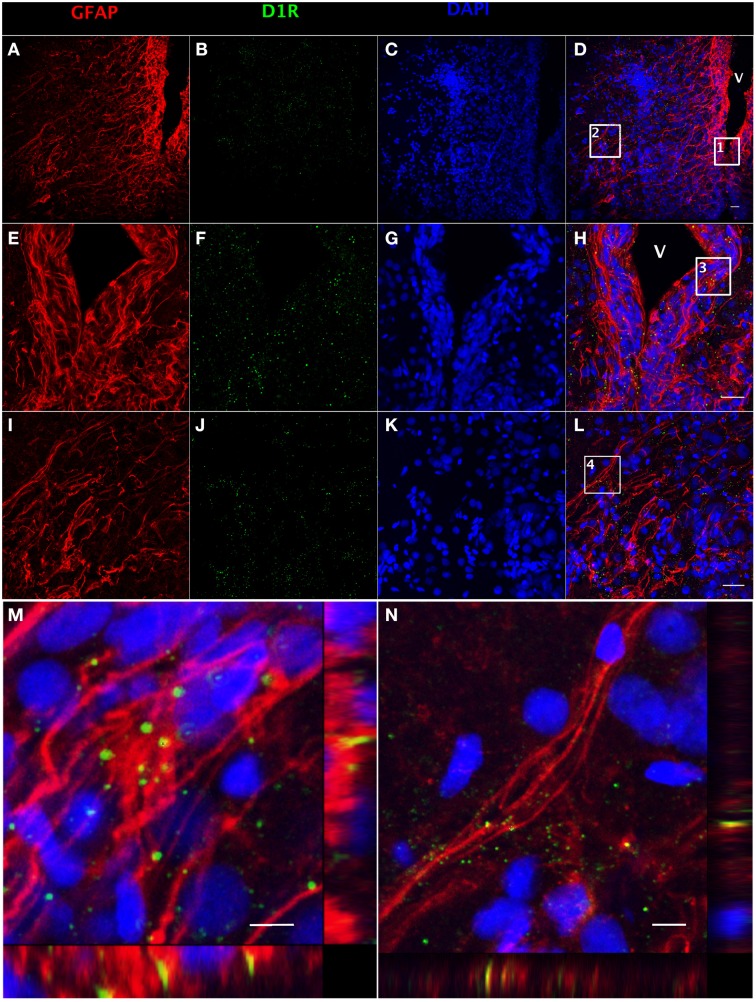
**Double fluorescence detection of GFAP (red) and D1R (green) in ventral telencephalic area of female goldfish brain**. The confocal image shows that RGCs marked by GFAP are located at the ventricular surface **(A,E)** with fibers extending to the area ventralis telencephali pars ventralis **(I)** and the expression of D1R at the ventricular surface and the area ventralis telencephali pars ventralis **(B,F,J)**. The expression of D1R by RGCs is shown in the merged images (V; ventricle, **D,H,L**). Magnification of the boxed area 1 in **(D)** shows that D1R is expressed by RGCs along the ventricular surface **(H)**. Magnification of the boxed area 2 in **(D)** shows that D1R is expressed by RGCs in the area ventralis telencephali pars ventralis **(L)**. Single slice scanning view of the boxed area 3 in panel H shows the colocalization of D1R and GFAP in RGCs along the ventricular surface **(M)**. Slice scan view of the boxed area 4 in **(L)** shows the colocalization of D1R and GFAP in RGCs in the area ventralis telencephali pars ventralis **(N)**. The nuclear stain DAPI (blue) is also shown **(C,G,K)**. Scale bar = 20 μm in **(A–L)**; Scale bar = 5 μm in **(M,N)**.

The presence of DA receptors on post-synaptic cells is required for direct regulation upon the release of DA by TH-positive catecholaminergic neurons. Both GFAP (Figure 3A) and dopamine D1 receptor (D1R) (Figure 3B)-positive staining can be observed along the ventricular surface (Figures 3E,F), and in the area ventralis telencephali pars ventralis (Figures 3I,J). The merged confocal images show the colocalization of GFAP and D1R immunoreactivity (Figures 3C,D,G,H,K–N), which indicates that RGCs express D1R. See Supplemental Figure 1 for validation of the D1R antibody (Supplemental Figure 1).

### Isolation and characterization of RGCs from goldfish brain

**Figure 4:**
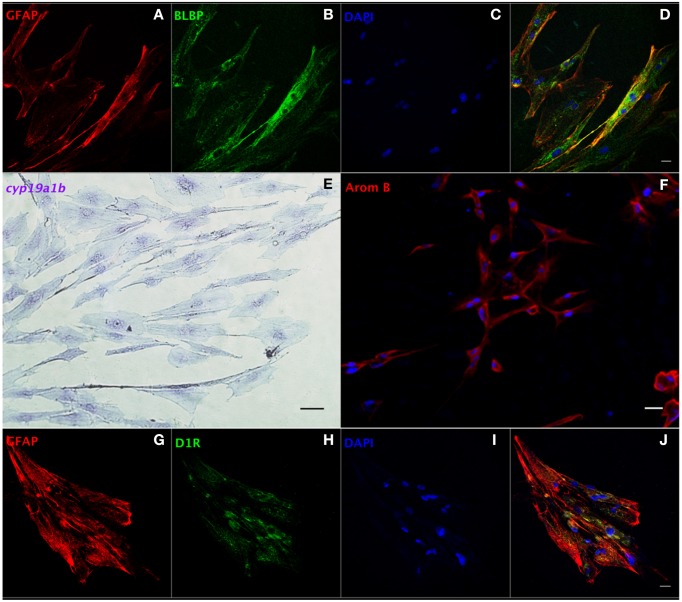
**Characterization of RGCs in primary culture by ***in situ*** hybridization and immunofluorescence**. Confocal image of double immunofluorescence labeling of GFAP (red, **A**) and BLBP (green, **B**) shows the colocalization of GFAP and BLBP in RGCs in culture **(D)**. Positive staining of *cyp19a1b* mRNA **(E)** and aromatase B protein (AromB) in RGC culture **(F)** indicates the expression of aromatase B in RGC cultures. Confocal imaging of double immunofluorescence labeling of GFAP (red, **G**) and D1R (green, **H**) shows the colocalization of GFAP and D1R in RGC culture **(J)**. The nuclear stain DAPI (blue) is also shown **(C,I)**. Scale bar = 10 μm.

We developed a protocol to isolate and culture RGCs from goldfish brain (Supplemental Information). Our method requires (a) trypsinization for tissue dissociation and subculture, (b) a complete cell culture medium with 15% FBS, and (c) changing the medium within a week after isolation. We propose this as an amenable technique since it yields a relatively pure RGC culture for functional studies, and a platform to screen potential drugs affecting RGCs. Both GFAP and brain lipid binding protein (BLBP) are highly expressed RGC markers *in vivo* (Forlano et al., 2001; Tong et al., 2009) and were used to identify cells in culture. At the third passage, most cells showed positive expression of GFAP (97%, Supplemental Figure 2A) and BLBP (98%, Supplemental Figure 2B) indicating that the cells maintained essential *in vivo* characteristics. The merged image indicates that all cells co-express GFAP and BLBP (Supplemental Figure 2D). Confocal images (Figures 4A–D) strengthen this conclusion and indicate that the majority of cells in culture are RGCs (>95%). Both *in situ* hybridization (Figure 4E) and immunofluorescence (Figure 4F) images indicate that RGCs in culture express *cyp19a1b* mRNA and aromatase B protein, further demonstrating the conservation of fundamental *in vivo* functions in culture conditions.

We next set out to determine the role of D1R in the regulation of aromatase B *in vitro*. Sequencing of a 205 bp nucleotide product of a specific PCR amplicon revealed identity (100%; Accession L08602.1) with goldfish D1R (Supplemental Figure 3A), and western blotting indicated that D1R was also translated and expressed in RGC cultures (Supplemental Figure 3B). Moreover, the yellow color in merged confocal images shows the colocalization of GFAP and D1R (Figures 4G–J). Following the establishment of both D1R and aromatase B in culture, we proceeded to determine the effects of a D1R agonist in aromatase B expression, as well as the signal transduction cascades associated with D1R activation and aromatase B expression.

### Dopamine D1 receptor activation regulates *cyp19a1b* mRNA in cultured RGCs

**Figure 5:**
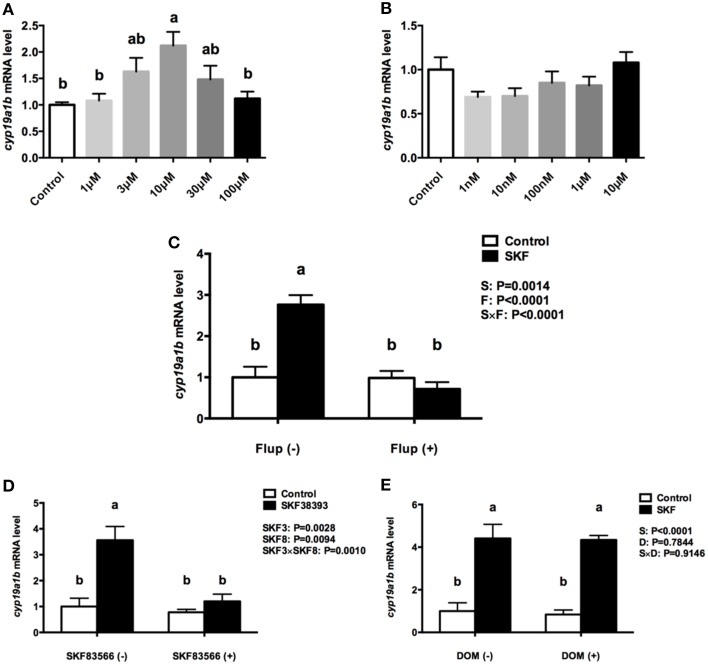
**The effects of dopaminergic compounds on ***cyp19a1b*** mRNA levels in RGC culture**. Quantitative real-time PCR analysis of the *cyp19a1b* mRNA, normalized to *18s* in primary RGC culture after 24 h exposure of different dosages of SKF 38393**(A)**, different dosages of Quinpirole **(B)**, SKF 38393 and/or Flup **(C)**, SKF 38393 and/or SKF 83566 **(D)**, SKF 38393 and/or DOM **(E)**. DMSO was used as a vehicle to dissolve SKF 83566 and DOM, which showed no effects on *cyp19a1b* mRNA levels in RGC culture (Supplemental Figure 4, *P* = 0.4153). Data were defined as fold change relative to control, the bars represent the mean + SEM (*n* = 4), each sample was analyzed in duplicate. a,b-Groups marked by different letters are significantly different (*P* < 0.05).

The RGC cultures were exposed to selective DA D1 (SKF 38393) or DA D2 (Quinpirole) agonists to investigate *cyp19a1b* mRNA regulation. After 24 h incubation with SKF 38393 in the 1–100 μM ranges, the qPCR data showed that 10 μM SKF 38393 effectively increased aromatase B mRNA by 2.1-fold (*P* = 0.007, Figure 5A). In contrast, the D2 agonist Quinpirole at 1, 10, 100 nM, 1 and 10 μM had no effect on *cyp19a1b* mRNA level (*P* = 0.12, Figure 5B). Based on the dose-response study, 10 μM SKF 38393 was chosen to investigate the specificity of aromatase B regulation in RGC culture. Firstly, the non-selective DA receptor antagonist, Flupentixol (Flup), was used to block DA receptors. Data showed that Flup alone did not affect *cyp19a1b* mRNA level (*P*>0.99), but the stimulatory effects of SKF 38393 on *cyp19a1b* mRNA (*P* < 0.0001) were completely inhibited (*P* = 0.81), indicating a receptor-dependent mechanism (Figure 5C). Secondly, the selective DA D1 receptor antagonist, SKF 83566 alone did not alter *cyp19a1b* mRNA (*P* = 0.9671), but the stimulatory effects of SKF 38393 on *cyp19a1b* mRNA (*P* = 0.001) were completely blocked by SKF 83566 (*P* = 0.82), indicating DA D1 receptor binding and activation involvement in *cyp19a1b* mRNA regulation (Figure 5D). Furthermore, it was confirmed that DA D2 receptor was not involved by using the selective DA D2 receptor antagonist Domperidone (DOM). Data showed that DOM alone did not have any effects on *cyp19a1b* mRNA (*P* = 0.99), and did not affect (*P* = 0.0003) SKF 38393 action (Figure 5E).

### Regulation of RGC *cyp19a1b* mRNA is through the D1R/cAMP/PKA/p-CREB signaling pathway

**Figure 6:**
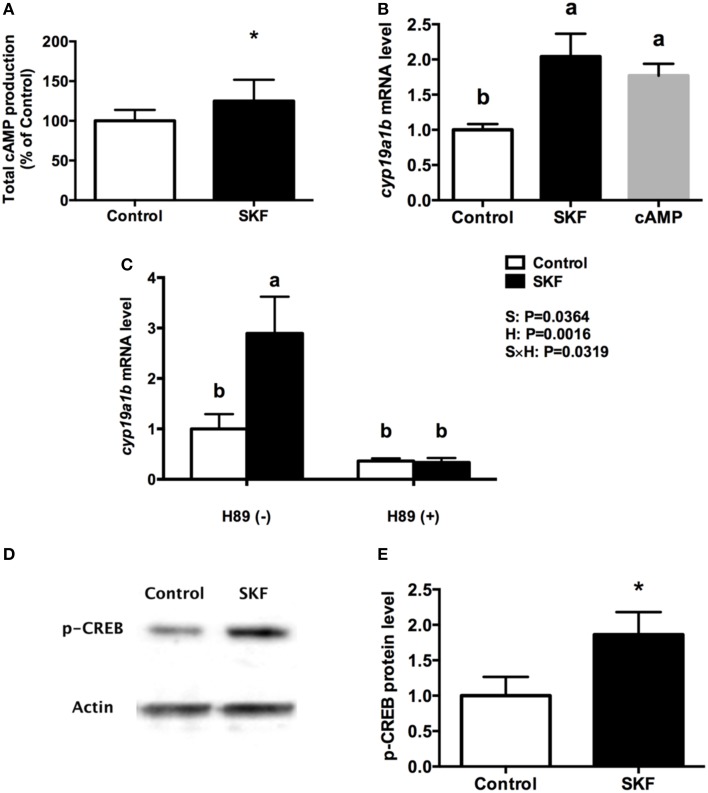
**Involvement of D1R/cAMP/PKA/p-CREB signaling pathway in dopaminergic regulation of ***cyp19a1b*** mRNA in RGC culture**. Total cAMP production rapidly increased by 10 μM SKF 38393 (30 min). Data were normalized to control value and defined as % of control, bars represent the mean + SEM (*n* = 3), and values for each sample were determined in duplicate. **(A)** Regulation of *cyp19a1b* mRNA in primary RGC culture after 24 h exposure of 8-Br-cAMP **(B)**, SKF 38393 and/or H89 **(C)**. Data were defined as fold change relative to control, bars represent the mean + SEM of *cyp19a1b* (*n* = 4), and values for each sample were determined in duplicate. Western blot image showing the effects of SKF 38393 on p-CREB immunoreactivity, where actin served as internal control **(D)**. The densitometric analysis of western blot is reported as an arbitrary value relative to the average of all bands on the same blot. Data were normalized and defined as fold change relative to control, and bars represent the mean + SEM (*n* = 6) **(E)**. a,b-Groups marked by different letters are significantly different (*P* < 0.05), ^*^Groups marked by asterisks are significantly different to control (*P* < 0.05).

Activation of D1R by SKF 38393 increased total cyclic adenosine monophosphate (cAMP) production by 25% (*P* = 0.03, Figure 6A). Furthermore, 10 μM 8-Br-cAMP increased *cyp19a1b* mRNA by 1.8-fold, similar to the 2-fold increases following 10 μM SKF 38393 (*P* = 0.02, Figure 6B). Therefore, we focused on the classic cAMP-dependent signaling pathway. The protein kinase A (PKA) inhibitor H89 blocked (*P* = 0.67) the D1R-dependent increase in *cyp19a1b* mRNA induced by SKF 38393 (*P* = 0.02) in cultured RGCs (Figure 6C). It is known that PKA activation leads to phosphorylation of cAMP response element binding protein (CREB). Exposure to SKF 38393 increased p-CREB protein levels by 1.8-fold (*P* = 0.0051, Figures 6D,E).

## Discussion

Together the results show that RGCs possess a functional D1R/cAMP/PKA/p-CREB signaling pathway that is associated with the control of aromatase B mRNA production. Immunohistochemical studies demonstrate that TH-positive catecholaminergic neurons are in close proximity to GFAP-positive RGCs expressing DA D1R, and raises the distinct possibility for dopaminergic regulation of RGCs. Dopamine may originate either directly from catecholaminergic neurons adjacent to RGCs as we have observed, or from the cerebrospinal fluid (CSF), because RGCs are positioned along the surface of the ventricle (Hornby and Piekut, 1990; Meek and Joosten, 1993; Smeets and González, 2000). It is well known that dopamine is present in CSF in rodents and humans (Fuxe et al., 2010). Studies in teleosts indicate that dopamine neurons in periventricular regions make contact with CSF in the third ventricle through their apical processes (Hornby and Piekut, 1990; Meek and Joosten, 1993; Smeets and González, 2000). These anatomical and biochemical data are strongly supported by D1R and GFAP colocalization in RGCs along the ventricular surface and in the area ventralis telencephali pars ventralis of female goldfish. This implicates D1R in the direct control of estrogen synthesis specifically in glial cells in the forebrain.

To develop the protocol for goldfish RGC culture system, we combined different primary glial cell culture protocols of mammalian astrocytes, fish astroglia, goldfish microglial cells, and zebrafish brainstem cells (Houalla and Levine, 2003; Crocker et al., 2008; Mack and Tiedemann, 2013; Tapanes-Castillo et al., 2014). There were several crucial differences between the protocols previously described and ours in order to optimize the isolation of RGCs. First, the trypsin dissociation was performed at RT instead of at 37°C for mammalian cells since higher temperature yielded much fewer adhered cells at day 4 after seeding. Considering the environmental temperature for goldfish, RT seems more reasonable and actually yielded better results with more adhered cells in culture flasks. Second, even though RGCs are extremely abundant in teleost brain compared to neurons (Sherwood et al., 2006; Reichenbach and Pannicke, 2008; Mack and Tiedemann, 2013), regular cell culture medium still could not completely deprive neurons in glial culture systems. Here a relatively higher percentage (15%) of FBS was used to increase the glial cell attachment to the dish and inhibit neuronal cell survival and growth, because certain components in serum, like glutamate, are toxic to neurons (Ye and Sontheimer, 1998). Furthermore, microglia and oligodendrocytes were removed and separated from the culture because of their differential adhesive properties compared to RGCs, as they need suitable cell culture medium supplements and take relatively longer time to adhere (Houalla and Levine, 2003). Oligodendrocytes and oligodendrocyte precursor cells are the least adhesive cell population in the brain, and can therefore be easily removed during changing of the cell culture medium (Itoh, 2002). According to Houalla et al., trypsin (0.25%) is toxic to goldfish microglial cells during the tissue dissociation step (Houalla and Levine, 2003), which was an advantage to our RGC culture development. However, trypsin solely is not enough for separating microglia from RGCs since in the first two passages some microglia-like cells appear on top of the RGC cell layer. This encouraged us to subculture and passage using trypsin to remove the existing microglia in culture. In summary, our protocol requires (a) trypsinization for tissue dissociation and subculture, (b) a complete cell culture medium with 15% FBS, and (c) changing the cell culture medium within a week after isolation. We propose this technique yields a relatively pure primary RGC culture for future characterization and other functional studies.

The first suggestion that dopamine may regulate aromatase in the brain came from studies in quail. There are catecholaminergic inputs close to aromatase-positive neurons, such as the sexually dimorphic medial preoptic nucleus and bed nucleus striae terminalis (Balthazart et al., 2002) but the mechanisms that are involved in this process remain unclear. Regardless, these pioneering studies indicated a relationship between dopamine receptors, aromatase and the control of sexual behavior in male birds (Balthazart et al., 2000; Absil et al., 2001; Ubuka et al., 2014).

The current study differs significantly from the earlier research because of the focus on neuron-RGC interactions. There were no prior *in vitro* studies on fish RGCs to allow investigation of direct neurotransmitter regulation of this critical cell type. An important advance is therefore the development and validation of the first RGC culture system for a teleost model. Our approach is efficient with at least 95% of the cells expressing major radial glial markers *in vitro* (Supplemental Figure 1). Markers for RGCs include GFAP, BLBP, vimentin, nestin, astrocyte-specific glutamate transporter (GLAST) and S100β (Forlano et al., 2005; Li et al., 2008; Brunne et al., 2010; Diotel et al., 2010b). There is no single marker that identifies all the RGCs in the teleost brain. Combinations of GFAP, BLBP and aromatase B are considered ideal (Verwer et al., 2007; Tong et al., 2009; Diotel et al., 2010a; Zupanc et al., 2012), and all were unequivocally present and colocalized in the cultured RGCs. The RGC cultures were also negative for Hu and zn-12 (Supplemental Figure 2), which are highly expressed in the nucleus and cell membrane of mature neurons, respectively. *In situ* hybridization, direct sequencing (Xing et al., 2014) and immunofluorescence were used to confirm the expression of *cyp19a1b* and production of aromatase B protein. The three independent techniques show that RGCs in culture are aromatase B-positive as they are *in vivo* (Forlano et al., 2001; Tong et al., 2009). Aromatase B is exclusively expressed by RGCs *in vivo* in fish (Forlano et al., 2001; Tong et al., 2009), and never in neurons as in mammals and birds (Azcoitia et al., 2011). Aromatase B is therefore a specific RGC protein with major roles in the control of neuroendocrine function and neurogenesis in teleost brain (Pellegrini et al., 2007).

Previous to this research the only known neuroendocrine factor up-regulating aromatase B in the teleost brain was estradiol itself (Forlano and Bass, 2005; Menuet et al., 2005; Diotel et al., 2010a), presumably from either peripheral sources, or by local conversion of testosterone. Teleost *cyp19a1b* genes contain functional estrogen response elements that explain these positive autoregulatory effects (Callard et al., 2001; Menuet et al., 2005; Diotel et al., 2010a). Immunocytochemistry, western blotting and direct sequencing all indicated the presence of D1R in goldfish RGCs. The D1R belongs to the G protein-coupled receptor superfamily, and activates adenylyl cyclase to promote cAMP accumulation (Kebabian and Calne, 1979). Previous studies identified the presence of a cAMP response element (CRE) located in the rat ovarian aromatase gene promoter (Hickey et al., 1990) and that aromatase gene transcription can be regulated by cAMP/CREB-dependent mechanisms (Carlone and Richards, 1997; Wang et al., 2012). Teleosts possess independent gonadal (*cyp19a1a*) and brain type (*cyp19a1b*) aromatases, which arose from a presumptive ancient genome duplication event in this lineage (Chiang et al., 2001). There are CRE-like sites located in *cyp19a1a* and *cyp19a1b* promoters of zebrafish and other species (Callard et al., 2001; Tchoudakova et al., 2001; Tong and Chung, 2003), which indicate that fish aromatases may be regulated by cAMP/CREB. We report that the levels of cAMP and p-CREB can be rapidly increased by SKF 38393. 8-Br-cAMP can stimulate *cyp19a1b* mRNA, and SKF 38393-stimulated *cyp19a1b* mRNA is inhibited by the PKA inhibitor H89. These results indicate that D1R activation via a cAMP/PKA/p-CREB pathway enhances aromatase B expression in RGCs.

The discovery that DA D1R activation stimulates aromatase B in RGCs is important for two main reasons: (A) it establishes that a catecholamine regulates a non-neuronal stem-like precursor cell that gives rise to both neurons and other glial cells (Schmechel and Rakic, 1979; Bentivoglio and Mazzarello, 1999; Zupanc and Clint, 2003; Zupanc et al., 2012), and (B) the data demonstrate that receptor activation regulates the estrogen synthesis enzyme aromatase B, which has many fundamental roles in brain differentiation, neural plasticity, neural regeneration, and neuroendocrine functions (Lephart, 1996; Azcoitia et al., 2003; Garcia-Segura et al., 2003; Ubuka et al., 2014). While it is well-known that estradiol stimulates neurogenesis in mammals (Garcia-Segura et al., 1999; Martínez-Cerdeño et al., 2006), emerging data in zebrafish indicate that estradiol inhibit neurogenesis (Diotel et al., 2013; Makantasi and Dermon, 2014). Moreover, chronic deprivation of estrogens by fadrozole inhibition of aromatase *in vivo* altered expression of numerous genes and pathways implicated in the control of neuronal plasticity and neuroendocrine function in female goldfish brain (Zhang et al., 2009), and include ependymins, synaptosomal-associated protein 25, neuropeptde Y, and pituitary adenylate cyclase-activating polypeptide, amongst others. By increasing *cyp19a1b* mRNA, DA D1R activation maintains the non-neuronal characteristic of estrogen synthesis in RGCs, and in turn these estrogens may signal locally to modulate adult neurogenesis. Together with recent data indicating an important role for dopamine in homeostasis and regeneration in the salamander brain (Berg et al., 2011), the involvement of neurotransmitters in controlling RGC function could be a plausible manner to link physiological variation or pathological loss of neuronal function to the need for repair of the adult CNS.

### Conflict of interest statement

The Reviewer Sophie Marie Croizier and Editor Sebastien G Bouret declare that, despite being affiliated to the same laboratory, and having collaborated on an article in 2014, the review was conducted objectively and no conflict of interest exists. The authors declare that the research was conducted in the absence of any commercial or financial relationships that could be construed as a potential conflict of interest.

## Supplementary material

Supplemental Figure 1 **Validation of D1R antibody**. The anti-D1R antibody against mouse and rat D1R was purchased from Acris Antibodies (AP09962PU-N). It was generated against a 19 amino acid sequence (IYRIAQKQIRRISALE) located in C-terminus region of D1R. This short amino acid sequence is 100% identical to an equivalent portion of goldfish D1R (accession no. P35406.1). D1R control peptide (Acris, AP09960CP-N) was purchased from Acris antibodies and preabsorbed (10 μg/ml at 4°C overnight) with D1R antibody. Immunohistochemistry was performed and showed no positive staining **(B)**. The nuclear stain DAPI (blue) is also shown **(A,C)**. Scale bar = 20 μm. To further test whether D1R antibody was recognizing D1R, western blot was performed to investigate the molecular weight of D1R antibody recognized protein. One band (40 kDa) was detectable using D1R antibody (1:1000) in goldfish telencephalon tissue (**D**, lane 1). No band was detected after perabsorbsion of D1R (1:1000) and control peptide (2.5 μg/ml at 4°C overnight) (**D**, lane 2).

Supplemental Figure 2 **Double immunofluorescence labeling of BLBP and GFAP, BLBP and Hu and BLBP and zn-12. (A,E,I)** BLBP labeling (green) of RGC in culture. **(B)** GFAP staining (red) in the same cells in **(A)**. **(F)** Hu staining (red) in the same cells in **(E)**. **(J)** zn-12 staining (red) in the same cells in **(I)**. **(C,G,K)** RGC nuclei were visualized by DAPI staining (blue). **(D,H,L)** Merged image shows expression of BLBP and GFAP, BLBP and Hu, BLBP and zn-12 in RGC primary culture. Scale bar = 20 μm. The percentage of positive GFAP and BLBP stained cells were counted out of total cells from 10 different views on one coverslip and six different coverslips with image J. Most cells in culture showed positive expression of GFAP (97 ± 0.4%, **A**) and BLBP (98 ± 0.3%, **B**). No immunoreactivity for Hu and zn-12 was detected.

Supplemental Figure 3 **The expression of D1R in RGC cultures**. Direct sequencing was performed using RGC cDNA samples to confirm the existence of D1R mRNA **(A)**, the PCR product is 100% identical to goldfish D1R (Accession L08602.1). Western blot image shows the expression of D1R in RGC culture (**B**, lane 1). No band was detected after preabsorption of D1R (1:1000) and control peptide (2.5 μg/ml at 4°C overnight) (**B**, lane 2).

Supplemental Figure 4 **The effects of DMSO on *cyp19a1b* mRNA levels in RGC culture**. Quantitative real-time PCR analysis showing the variations in the relative amounts of the *cyp19a1b* mRNA to *18s* (A) in primary RGC culture after 24 h exposure of DMSO (0.1%). Data show no effects of DMSO on *cyp19a1b* mRNA levels in RGC culture (*P* = 0.42).
