# Supplementary figures and images for: Dopamine D1 receptor activation regulates the expression of the estrogen synthesis gene aromatase B in radial glial cells
Source: Front Neurosci. 2015 Sep 2;9:310. doi: 10.3389/fnins.2015.00310 (PMC4557113; doi:10.3389/fnins.2015.00310)

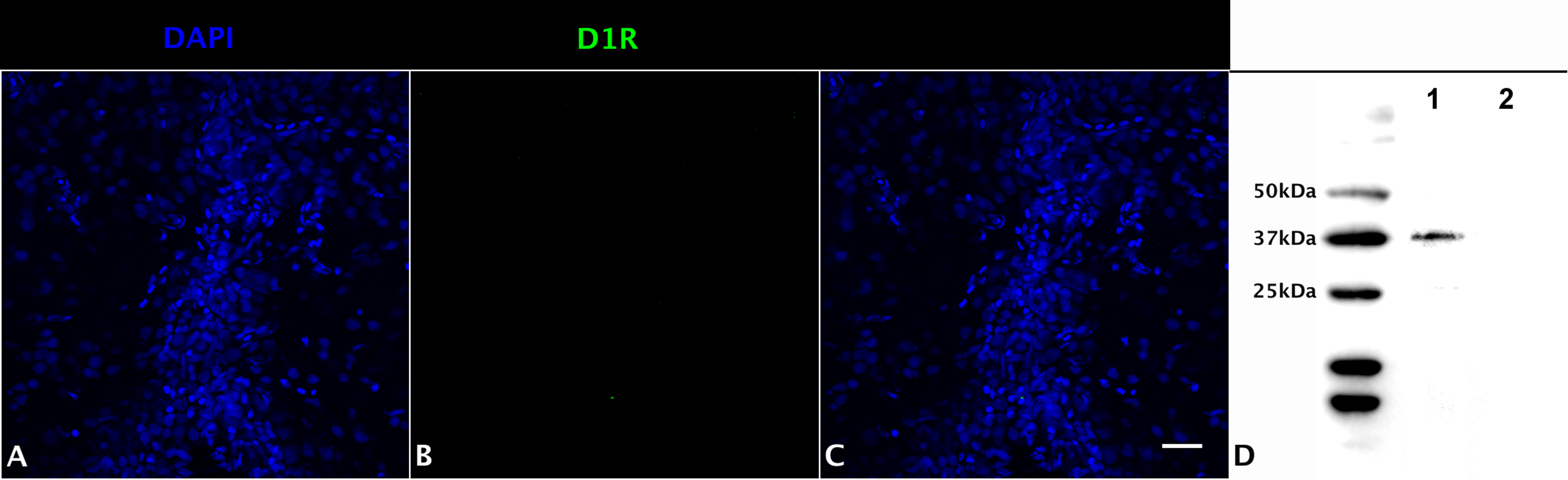

Supplement: Supplemental Figure 1 — Validation of D1R antibody. The anti-D1R antibody against mouse and rat D1R was purchased from Acris Antibodies (AP09962PU-N). It was generated against a 19 amino acid sequence (IYRIAQKQIRRISALE) located in C-terminus region of D1R. This short amino acid sequence is 100% identical to an equivalent portion of goldfish D1R (accession no. P35406.1). D1R control peptide (Acris, AP09960CP-N) was purchased from Acris antibodies and preabsorbed (10 μg/ml at 4°C overnight) with D1R antibody. Immunohistochemistry was performed and showed no positive staining (B). The nuclear stain DAPI (blue) is also shown (A,C). Scale bar = 20 μm. To further test whether D1R antibody was recognizing D1R, western blot was performed to investigate the molecular weight of D1R antibody recognized protein. One band (40 kDa) was detectable using D1R antibody (1:1000) in goldfish telencephalon tissue (D, lane 1). No band was detected after perabsorbsion of D1R (1:1000) and control peptide (2.5 μg/ml at 4°C overnight) (D, lane 2). [file Image1.TIF]

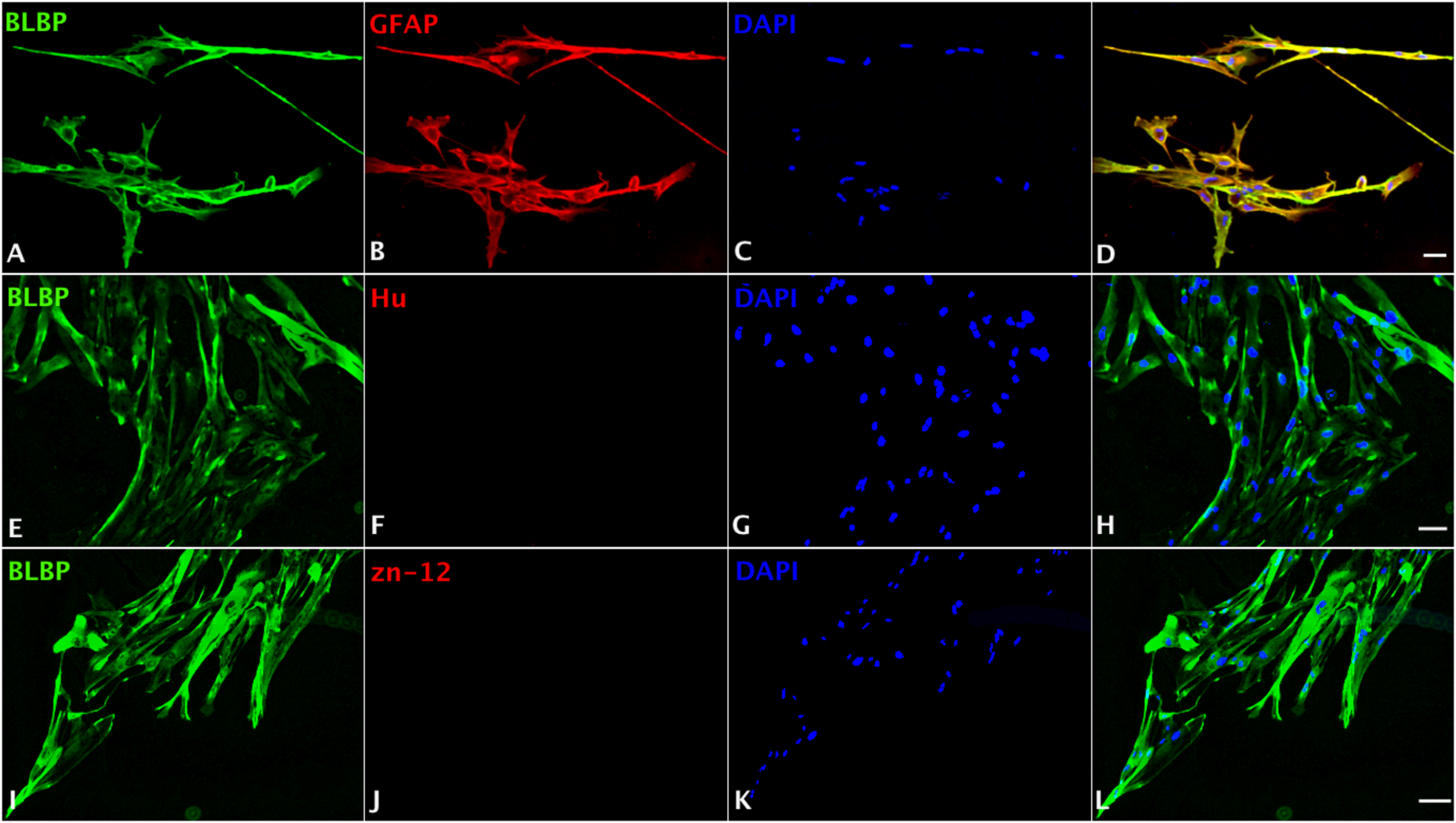

Supplement: Supplemental Figure 2 — Double immunofluorescence labeling of BLBP and GFAP, BLBP and Hu and BLBP and zn-12. (A,E,I) BLBP labeling (green) of RGC in culture. (B) GFAP staining (red) in the same cells in (A). (F) Hu staining (red) in the same cells in (E). (J) zn-12 staining (red) in the same cells in (I). (C,G,K) RGC nuclei were visualized by DAPI staining (blue). (D,H,L) Merged image shows expression of BLBP and GFAP, BLBP and Hu, BLBP and zn-12 in RGC primary culture. Scale bar = 20 μm. The percentage of positive GFAP and BLBP stained cells were counted out of total cells from 10 different views on one coverslip and six different coverslips with image J. Most cells in culture showed positive expression of GFAP (97 ± 0.4%, A) and BLBP (98 ± 0.3%, B). No immunoreactivity for Hu and zn-12 was detected. [file Image2.TIF]

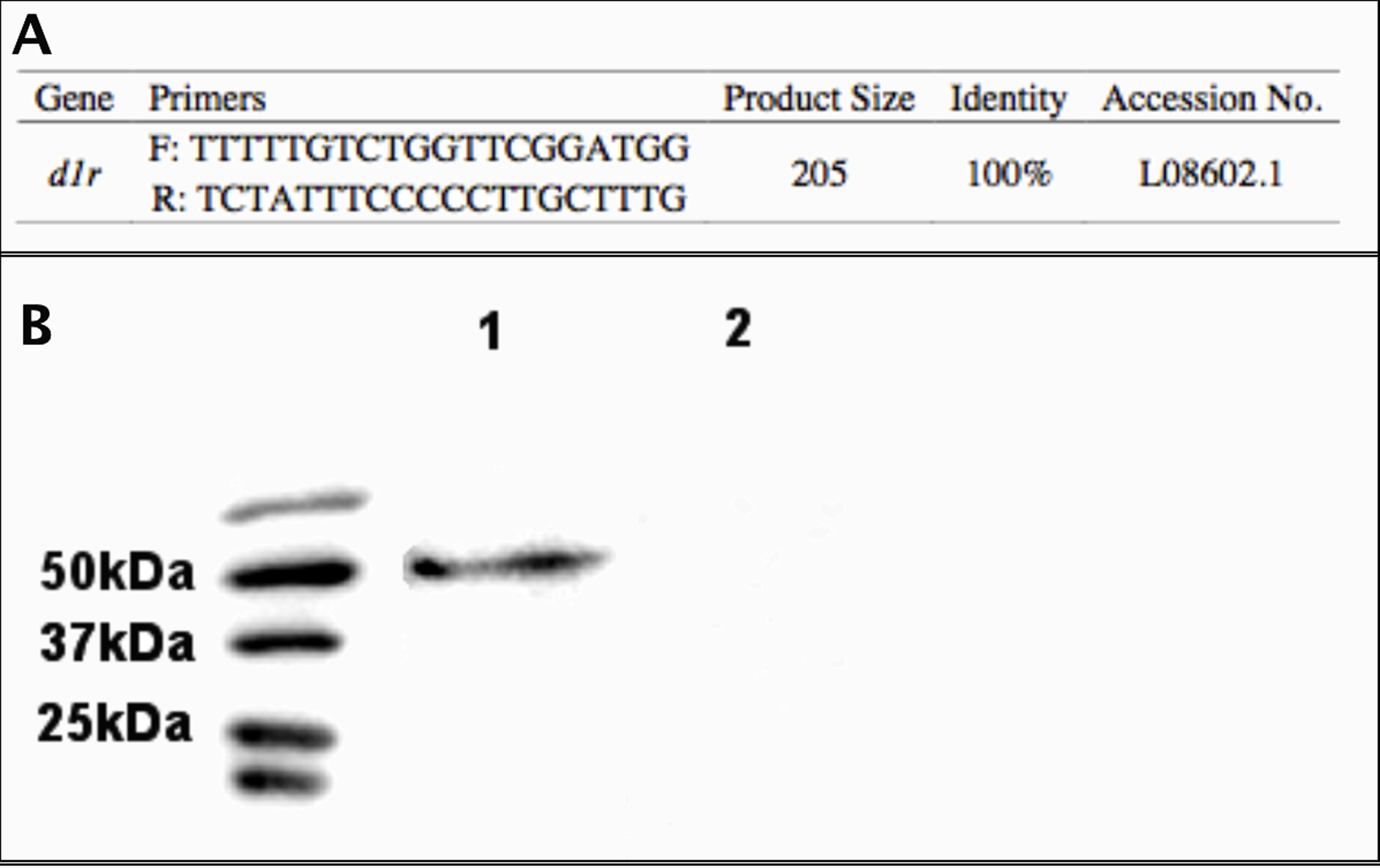

Supplement: Supplemental Figure 3 — The expression of D1R in RGC cultures. Direct sequencing was performed using RGC cDNA samples to confirm the existence of D1R mRNA (A), the PCR product is 100% identical to goldfish D1R (Accession L08602.1). Western blot image shows the expression of D1R in RGC culture (B, lane 1). No band was detected after preabsorption of D1R (1:1000) and control peptide (2.5 μg/ml at 4°C overnight) (B, lane 2). [file Image3.TIF]

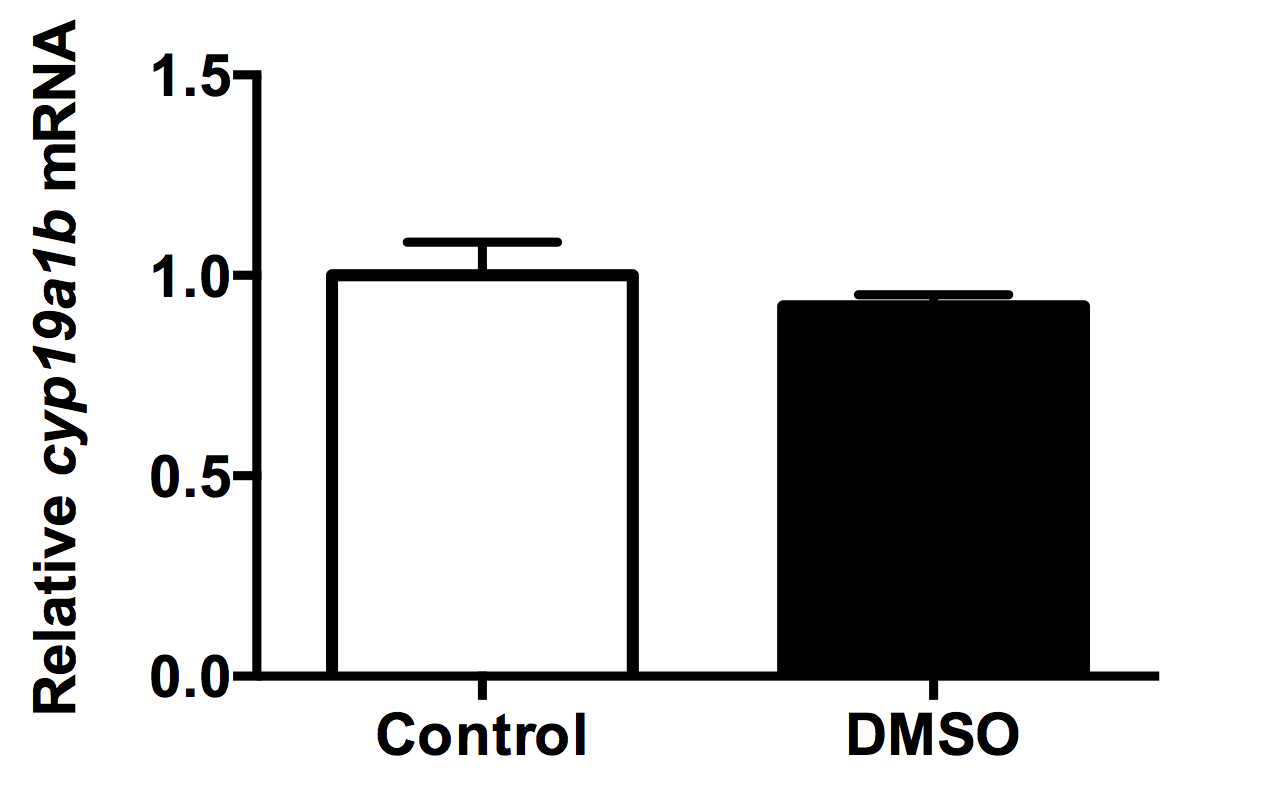

Supplement: Supplemental Figure 4 — The effects of DMSO on cyp19a1b mRNA levels in RGC culture. Quantitative real-time PCR analysis showing the variations in the relative amounts of the cyp19a1b mRNA to 18s (A) in primary RGC culture after 24 h exposure of DMSO (0.1%). Data show no effects of DMSO on cyp19a1b mRNA levels in RGC culture (P = 0.42). [file Image4.TIFF]
